# Supplementary material for: Gα13 restricts nutrient driven proliferation in mucosal germinal centers
Source: Nat Immunol. 2024 Jul 18;25(9):1718–30. doi: 10.1038/s41590-024-01910-0 (PMC11362015; doi:10.1038/s41590-024-01910-0)
Supplement: Supplementary file 1 — Reporting Summary [file 41590_2024_1910_MOESM1_ESM.pdf]

nature portfolio

Corresponding author(s): Jagan Muppidi

Last updated by author(s): Jun 23, 2024

# Reporting Summary

Nature Portfolio wishes to improve the reproducibility of the work that we publish. This form provides structure for consistency and transparency in reporting. For further information on Nature Portfolio policies, see our Editorial Policies and the Editorial Policy Checklist.

## Statistics

For all statistical analyses, confirm that the following items are present in the figure legend, table legend, main text, or Methods section.

| n/a | Confirmed | |
|---|---|---|
| ☐ | ☒ | The exact sample size (*n*) for each experimental group/condition, given as a discrete number and unit of measurement |
| ☐ | ☒ | A statement on whether measurements were taken from distinct samples or whether the same sample was measured repeatedly |
| ☐ | ☒ | The statistical test(s) used AND whether they are one- or two-sided<br>*Only common tests should be described solely by name; describe more complex techniques in the Methods section.* |
| ☒ | ☐ | A description of all covariates tested |
| ☐ | ☒ | A description of any assumptions or corrections, such as tests of normality and adjustment for multiple comparisons |
| ☐ | ☒ | A full description of the statistical parameters including central tendency (e.g. means) or other basic estimates (e.g. regression coefficient) AND variation (e.g. standard deviation) or associated estimates of uncertainty (e.g. confidence intervals) |
| ☐ | ☒ | For null hypothesis testing, the test statistic (e.g. *F*, *t*, *r*) with confidence intervals, effect sizes, degrees of freedom and *P* value noted<br>*Give P values as exact values whenever suitable.* |
| ☒ | ☐ | For Bayesian analysis, information on the choice of priors and Markov chain Monte Carlo settings |
| ☒ | ☐ | For hierarchical and complex designs, identification of the appropriate level for tests and full reporting of outcomes |
| ☒ | ☐ | Estimates of effect sizes (e.g. Cohen's *d*, Pearson's *r*), indicating how they were calculated |

*Our web collection on statistics for biologists contains articles on many of the points above.*

## Software and code

Policy information about availability of computer code

| | |
|---|---|
| Data collection | Flow cytometry data were collected with CytExpert v2.3.<br>IF data was collected with LASX v4.5<br>IHC and H&E data were collected with Zen v3.8.<br>Western blot images were collected with Image Lab Touch Software v3.01 (BioRad).<br>CRISPR screen reads were generated using NextSeq 1000/2000 Control Software (v. 1.2.036376) (Illumina) and demultiplexed using DRAGEN (v.3.7.4) (Illumina). |
| Data analysis | Flow cytometry data were analyzed using FlowJo v10.<br>Imaging data were processed and analyzed using Imaris v10 and FlowJo v10.<br>Single cell sequencing files were processed and aligned to mm10, and count matrices were generated using Cell Ranger (v6.0.0). Further analyses were performed in R using the Seurat package (v4). Gene set enrichment analysis was done using the FGSEA workflow.<br>Data analysis was performed on Microsoft Excel v16.80 and Prism v10.<br>GSEA software (v4.0.3)<br>Western blot images were analyzed with Image Lab v6.1 (Bio-Rad).<br>CRISPR screen reads were extracted from fastq files and normalized using custom perl scripts and Bowtie2 (version 2.2.9). |

For manuscripts utilizing custom algorithms or software that are central to the research but not yet described in published literature, software must be made available to editors and reviewers. We strongly encourage code deposition in a community repository (e.g. GitHub). See the Nature Portfolio guidelines for submitting code & software for further information.

## Data

Policy information about availability of data

All manuscripts must include a data availability statement. This statement should provide the following information, where applicable:

- Accession codes, unique identifiers, or web links for publicly available datasets
- A description of any restrictions on data availability
- For clinical datasets or third party data, please ensure that the statement adheres to our policy

Cell line and single cell RNA seq data was uploaded to the Gene Expression Omnibus (GEO) under accession number GSE253435.
Repertoire sequencing data and CRISPR screen score (CSS) data used in this study are provided in Figshare (https://figshare.com/s/dc9ea731f2c72b19b4f3)
Source data are provided with this paper.

## Research involving human participants, their data, or biological material

Policy information about studies with human participants or human data. See also policy information about sex, gender (identity/presentation), and sexual orientation and race, ethnicity and racism.

| | |
|---|---|
| Reporting on sex and gender | Not applicable |
| Reporting on race, ethnicity, or other socially relevant groupings | Not applicable |
| Population characteristics | Not applicable |
| Recruitment | Not applicable |
| Ethics oversight | Not applicable |

Note that full information on the approval of the study protocol must also be provided in the manuscript.

# Field-specific reporting

Please select the one below that is the best fit for your research. If you are not sure, read the appropriate sections before making your selection.

☒ Life sciences ☐ Behavioural & social sciences ☐ Ecological, evolutionary & environmental sciences

For a reference copy of the document with all sections, see nature.com/documents/nr-reporting-summary-flat.pdf

# Life sciences study design

All studies must disclose on these points even when the disclosure is negative.

| | |
|---|---|
| Sample size | No statistical methods were used to pre-determine sample sizes, but our sample sizes are similar to those reported in previous publicationsn (Refs. 6, 9, 14, 53). Sample size was based on previous experiments and were of sufficient size to detect differences between groups and were guided by the 3R principles to reduce animal numbers. |
| Data exclusions | No data were excluded from analysis. |
| Replication | Biological and technical replicates were performed. Most of the experiments have been reproduced 2-3 independent times with comparable results. In some instances, data were pooled from independent experiments. Information on replicates is included in figure legends. |
| Randomization | Groups were allocated on the basis of genotype. Littermates that were also sex-matched were used whenever possible to control for co-variates. For experiments with treatment, mice were allocated to control and experimental groups randomly. |
| Blinding | Investigators were not blinded to mouse genotypes or sample type during data collection. Blinding was not feasible due to the staffing requirements that would be needed for blinded experiment and the need for cage labeling in the colony. Data reported are based primarily on quantitative flow cytometry and image quantification analysis which are not subject to the same potential biases as experiments with more subjective readouts. |

# Reporting for specific materials, systems and methods

We require information from authors about some types of materials, experimental systems and methods used in many studies. Here, indicate whether each material, system or method listed is relevant to your study. If you are not sure if a list item applies to your research, read the appropriate section before selecting a response.

## Materials & experimental systems

| n/a | Involved in the study |
|---|---|
| ☐ | ☒ Antibodies |
| ☐ | ☒ Eukaryotic cell lines |
| ☒ | ☐ Palaeontology and archaeology |
| ☐ | ☒ Animals and other organisms |
| ☒ | ☐ Clinical data |
| ☒ | ☐ Dual use research of concern |
| ☒ | ☐ Plants |

## Methods

| n/a | Involved in the study |
|---|---|
| ☒ | ☐ ChIP-seq |
| ☐ | ☒ Flow cytometry |
| ☒ | ☐ MRI-based neuroimaging |

# Antibodies

Antibodies used

Antibodies used for Flow cytometry:
Fixable Viability Dye eFluor 780 (ebiosciences) 1:1000
BV786 or BUV395–conjugated anti-B220 (RA3-6B2; BD), 1:400
BUV395-conjugated anti-CD4 (RM4-5; BD), 1:400
Pacific blue or Alexa Fluor 647–conjugated GL7 (GL-7; BioLegend), 1:400
BV650 conjugated anti-IgD (11- 26c.2a; BioLegend), 1:400
PerCP Cy5.5 or PE-Cy7–conjugated anti-CD38 (90; BioLegend), 1:400
PE-Cy7, PE or BV421-conjugated anti-Fas (Jo2; BD), 1:400
FITC, PerCP-Cy5.5 or Alexa Flour 700-conjugated anti-CD45.2 (104; BioLegend), 1:400
PE-Cy7 or PerCP-Cy5.5–conjugated anti-CD45.1 (A20; BioLegend), 1:400
BV786–conjugated anti-CD86 (GL-1; BioLegend), 1:100
PE-conjugated, APC- conjugated anti-CXCR4 (2B11; ebiosciences), 1:100
Alexa Fluor 647–conjugated anti-c-Myc (Y69; Abcam), 1:400
PE-conjugated anti-Cyclin D3 (DCS-22; Biolegened), 1:400
FITC or APC conjugated anti-BrdU (BU20A; eBioscience), 1:100
p-AKT S473 (D9E; Cell Signaling; 1:400)
pRPS6 Ser240/244 (D68F8; Cell Signaling) 1:400
AF647-conjugated anti-rabbit IgG (Invitrogen)1:1000

For Immunohistochemistry experiments:
biotinylated anti-GL7 (GL7; Biolegend), 1:400
biotinylated anti-B220 (RA3-6B2; Biolegend), 1:200
biotinylated anti-CD35 (8C12; BD Biosciences), 1:200
unlabeled polyclonal goat anti-mouse IgD (Cedarlane) 1:1000

For Immunofluorescence experiments
SparkRed 718-conjugated anti-B220 (RA3-6B2; BioLegend) 1:200
AF488-conjugated anti-IgD (11-26c.2a; BioLegend) 1:100
AF647–conjugated GL7 (GL-7; BioLegend) 1:100
biotin-conjugated anti-CD35 (8C12; BD) 1:200
biotin-conjugated anti-LYVE1 (ALY7, eBioscience) 1:200
rabbit anti-Myc (D3N8F; Cell Signaling) 1:400
rabbit anti-phospho-ribosomal protein S6 (pRPS6) (Ser240/244) (D68F8; Cell Signaling) 1:800
BV421-conjugated streptavidin (BD) 1:400
AF555-conjugated anti-rabbit IgG (Invitrogen)1:200
JOPRO-1 (Invitrogen) 1:10,000

For Western blot experiments:
MYC (D3N8F; Cell Signaling; 1:2000)
CCND3 (DCS22; Cell Signaling; 1:2000)
Actin (13E5; Cell Signaling; 1:4000)
p-P70S6K T389 (108D2; Cell Signaling; 1:2000)
P70S6K (polyclonal rabbit antibody #9202; Cell Signaling; 1:2000)
p-RPS6 S235/6 (D57.2.2E; Cell Signaling; 1:4000)
RPS6 (5G10; Cell Signaling; 1:4000)
p-AKT S473 (D9E; Cell Signaling; 1:4000)
p-AKT T308 (D25E6; Cell Signaling; 1:4000)
p-CCND3 T283 (E1V6W; Cell Signaling; 1:2000)

Validation

All antibodies used in this study are from widely used commercial sources and have been previously described in the literature and validated by the vendors. Validation data and citation information are available on the manufacturer's website (links below). Appropriate antibody dilutions were performed based on preliminary experiments and intensity of fluorescent signals. Dilutions for flow cytometry antibodies are referred to a staining volume of 40 ul per sample (~1 × 106 cells).

Antibodies used for Flow cytometry:
Fixable Viability Dye eFluor 780 (ebiosciences) 1:1000
https://www.thermofisher.com/order/catalog/product/65-0865-14

BV786 or BUV395–conjugated anti-B220 (RA3-6B2; BD), 1:400
https://www.bdbiosciences.com/en-us/products/reagents/flow-cytometry-reagents/research-reagents/single-color-antibodies-ruo/buv395-rat-anti-mouse-cd45r-b220.563793
https://www.bdbiosciences.com/en-us/products/reagents/flow-cytometry-reagents/research-reagents/single-color-antibodies-ruo/bv786-rat-anti-mouse-cd45r-b220.563894

BUV395-conjugated anti-CD4 (RM4-5; BD), 1:400
https://www.bdbiosciences.com/en-us/products/reagents/flow-cytometry-reagents/research-reagents/single-color-antibodies-ruo/buv395-rat-anti-mouse-cd4.568375

Pacific blue or Alexa Fluor 647–conjugated GL7 (GL-7; BioLegend), 1:400
https://www.biolegend.com/en-gb/products/pacific-blue-anti-mouse-human-gl7-antigen-t-and-b-cell-activation-marker-antibody-9580
https://www.biolegend.com/en-gb/products/alexa-fluor-647-anti-mouse-human-gl7-antigen-t-and-b-cell-activation-marker-antibody-8602

BV650 conjugated anti-IgD (11- 26c.2a; BioLegend), 1:400
https://www.biolegend.com/en-gb/products/brilliant-violet-650-anti-mouse-igd-9031

PerCP Cy5.5 or PE-Cy7–conjugated anti-CD38 (90; BioLegend), 1:400
https://www.biolegend.com/en-gb/products/pe-cyanine7-anti-mouse-cd38-antibody-3926
https://www.biolegend.com/en-gb/products/percp-cyanine5-5-anti-mouse-cd38-antibody-9563

PE-Cy7, PE or BV421-conjugated anti-Fas (Jo2; BD), 1:400
https://www.bdbiosciences.com/en-us/products/reagents/flow-cytometry-reagents/research-reagents/single-color-antibodies-ruo/bv421-hamster-anti-mouse-cd95.562633
https://www.bdbiosciences.com/en-us/products/reagents/flow-cytometry-reagents/research-reagents/single-color-antibodies-ruo/pe-cy-7-hamster-anti-mouse-cd95.557653
https://www.bdbiosciences.com/en-us/products/reagents/flow-cytometry-reagents/research-reagents/single-color-antibodies-ruo/pe-hamster-anti-mouse-cd95.554258

FITC, PerCP-Cy5.5 or Alexa Flour 700-conjugated anti-CD45.2 (104; BioLegend), 1:400
https://www.biolegend.com/en-gb/products/fitc-anti-mouse-cd45-2-antibody-6
https://www.biolegend.com/en-gb/products/percp-cyanine5-5-anti-mouse-cd452-antibody-4271
https://www.biolegend.com/en-gb/products/alexa-fluor-700-anti-mouse-cd45-2-antibody-3393

PE-Cy7 or PerCP-Cy5.5–conjugated anti-CD45.1 (A20; BioLegend), 1:400
https://www.biolegend.com/en-gb/products/pe-cyanine7-anti-mouse-cd45-1-antibody-4917
https://www.biolegend.com/en-gb/products/percp-cyanine5-5-anti-mouse-cd45-1-antibody-4269

BV786–conjugated anti-CD86 (GL-1; BioLegend), 1:100
https://www.biolegend.com/en-gb/products/brilliant-violet-785-anti-mouse-cd86-antibody-12818

PE-conjugated, APC- conjugated anti-CXCR4 (2B11; ebiosciences), 1:100
https://www.thermofisher.com/antibody/product/CD184-CXCR4-Antibody-clone-2B11-Monoclonal/12-9991-82
https://www.thermofisher.com/antibody/product/CD184-CXCR4-Antibody-clone-2B11-Monoclonal/17-9991-82?imageId=1023016

Alexa Fluor 647–conjugated anti-c-Myc (Y69; Abcam), 1:400
https://www.abcam.com/products/primary-antibodies/alexa-fluor-647-c-myc-antibody-y69-ab190560.html

PE-conjugated anti-Cyclin D3 (DCS-22; Biolegend), 1:400
https://www.biolegend.com/en-gb/products/pe-anti-cyclin-d3-antibody-14405

FITC or APC conjugated anti-BrdU (BU20A; eBioscience), 1:100
https://www.thermofisher.com/antibody/product/BrdU-Antibody-clone-BU20A-Monoclonal/11-5071-42
https://www.thermofisher.com/antibody/product/BrdU-Antibody-clone-BU20A-Monoclonal/17-5071-42

p-AKT S473 (D9E; Cell Signaling; 1:400)
https://www.cellsignal.com/products/primary-antibodies/phospho-akt-ser473-d9e-xp-rabbit-mab/4060

pRPS6 Ser240/244 (D68F8; Cell Signaling) 1:400
https://www.cellsignal.com/products/primary-antibodies/phospho-s6-ribosomal-protein-ser240-244-d68f8-xp-rabbit-mab/5364

AF647-conjugated anti-rabbit IgG (Invitrogen)1:1000
https://www.thermofisher.com/antibody/product/Goat-anti-Rabbit-IgG-H-L-Highly-Cross-Adsorbed-Secondary-Antibody-Polyclonal/A-21245

For Immunohistochemistry experiments:
biotinylated anti-GL7 (GL7; Biolegend), 1:400
https://www.biolegend.com/en-gb/products/biotin-anti-mousehuman-gl7-antigen-t-and-b-cell-activation-marker-antibody-15161

biotinylated anti-B220 (RA3-6B2; Biolegend), 1:200
https://www.biolegend.com/en-gb/products/biotin-anti-mouse-human-cd45r-b220-antibody-444

biotinylated anti-CD35 (8C12; BD Biosciences), 1:200
https://www.bdbiosciences.com/en-us/products/reagents/flow-cytometry-reagents/research-reagents/single-color-antibodies-ruo/

biotin-rat-anti-mouse-cd35.553816

unlabeled polyclonal goat anti-mouse IgD (Cedarlane) 1:1000
https://www.cedarlanelabs.com

For Immunofluorescence experiments
SparkRed 718-conjugated anti-B220 (RA3-6B2; BioLegend) 1:200
https://www.biolegend.com/en-gb/products/spark-red-718-anti-mouse-human-cd45r-b220-antibody-22290

AF488-conjugated anti-IgD (11-26c.2a; BioLegend) 1:100
https://www.biolegend.com/en-gb/products/alexa-fluor-488-anti-mouse-igd-7092

AF647–conjugated GL7 (GL-7; BioLegend) 1:100
https://www.biolegend.com/en-gb/products/alexa-fluor-647-anti-mouse-human-gl7-antigen-t-and-b-cell-activation-marker-antibody-8602

biotin-conjugated anti-CD35 (8C12; BD) 1:200
https://www.bdbiosciences.com/en-us/products/reagents/flow-cytometry-reagents/research-reagents/single-color-antibodies-ruo/biotin-rat-anti-mouse-cd35.553816

biotin-conjugated anti-LYVE1 (ALY7, eBioscience) 1:200
https://www.thermofisher.com/antibody/product/LYVE1-Antibody-clone-ALY7-Monoclonal/13-0443-82

rabbit anti-Myc (D3N8F; Cell Signaling) 1:400
https://www.cellsignal.com/products/primary-antibodies/c-myc-n-myc-d3n8f-rabbit-mab/13987

rabbit anti-phospho-ribosomal protein S6 (pRPS6) (Ser240/244) (D68F8; Cell Signaling) 1:800
https://www.cellsignal.com/products/primary-antibodies/phospho-s6-ribosomal-protein-ser240-244-d68f8-xp-rabbit-mab/5364

BV421-conjugated streptavidin (BD) 1:400
https://www.bdbiosciences.com/en-us/products/reagents/flow-cytometry-reagents/research-reagents/single-color-antibodies-ruo/bv421-streptavidin.563259

AF555-conjugated anti-rabbit IgG (Invitrogen)1:200
https://www.thermofisher.com/antibody/product/Goat-anti-Rabbit-IgG-H-L-Cross-Adsorbed-Secondary-Antibody-Polyclonal/A-21428

JOPRO-1 (Invitrogen) 1:10,000
https://www.thermofisher.com/order/catalog/product/Y3603

For Western blot experiments:
MYC (D3N8F; Cell Signaling; 1:2000)
https://www.cellsignal.com/products/primary-antibodies/c-myc-n-myc-d3n8f-rabbit-mab/13987

CCND3 (DCS22; Cell Signaling; 1:2000)
https://www.cellsignal.com/products/primary-antibodies/cyclin-d3-dcs22-mouse-mab/2936

Actin (13E5; Cell Signaling; 1:4000)
https://www.cellsignal.com/products/primary-antibodies/b-actin-13e5-rabbit-mab/4970

p-P70S6K T389 (108D2; Cell Signaling; 1:2000)
https://www.cellsignal.com/products/primary-antibodies/phospho-p70-s6-kinase-thr389-108d2-rabbit-mab/9234

P70S6K (polyclonal rabbit antibody #9202; Cell Signaling; 1:2000)
https://www.cellsignal.com/products/primary-antibodies/p70-s6-kinase-antibody/9202

p-RPS6 S235/6 (D57.2.2E; Cell Signaling; 1:4000)
https://www.cellsignal.com/products/primary-antibodies/phospho-s6-ribosomal-protein-ser235-236-d57-2-2e-xp-rabbit-mab/4858

RPS6 (5G10; Cell Signaling; 1:4000)
https://www.cellsignal.com/products/primary-antibodies/s6-ribosomal-protein-5g10-rabbit-mab/2217

p-AKT S473 (D9E; Cell Signaling; 1:4000)
https://www.cellsignal.com/products/primary-antibodies/phospho-akt-ser473-d9e-xp-rabbit-mab/4060

p-AKT T308 (D25E6; Cell Signaling; 1:4000)
https://www.cellsignal.com/products/primary-antibodies/phospho-akt-thr308-d25e6-xp-rabbit-mab/13038

p-CCND3 T283 (E1V6W; Cell Signaling; 1:2000)
https://www.cellsignal.com/products/primary-antibodies/phospho-cyclin-d3-thr283-e1v6w-rabbit-mab/53966

## Eukaryotic cell lines

Policy information about cell lines and Sex and Gender in Research

| | |
|---|---|
| Cell line source(s) | NUDUL1 Human diffuse large B cell line ATCC CRL-2969<br>OCI-Ly8 Human diffuse large B cell line NCI<br>Dogkit Human diffuse large B cell line DMSZ<br>The Platinum E (Plat-E ) retroviral packaging cell line was a gift from Susan R. Schwab at New York University.<br>293FT Transformed Human kidney cell line Thermo Fisher R70007 |
| Authentication | We used a "DNA fingerprinting" method to test for the presence or absence of 16 common copy number variants allowing the detection of cross-contamination. |
| Mycoplasma contamination | All cell line tested negative for mycoplasma using using the MycoAlert Mycoplasma Detection Kit (Lonza). Cell lines were tested regularly and preventative treatment was undertaken using MycoZap (Lonza) and Plasmocin (InvivoGen). |
| Commonly misidentified lines<br>(See ICLAC register) | No cell lines used in this study among commonly misidentified lines. |

## Animals and other research organisms

Policy information about studies involving animals; ARRIVE guidelines recommended for reporting animal research, and Sex and Gender in Research

| | |
|---|---|
| Laboratory animals | Male and female mus musculus were used.<br>In bone marrow chimeras experiments, animals were irradiated between 6 and 10 weeks of age and analyzed 7-10 weeks following irradiation (Fig. 1e,f,g, Fig. 2, Fig. 3e-g, j-l, Fig. 4l, Fig. 6b-e, g-h, k-l, Fig. 7d-f, ED Fig. 2b-f, ED Fig. 3, ED Fig. 4i-j, ED Fig. 6a-c, e,g, ED Fig. 7b-d, g h-i) . Most other experiments were performed on adult animals between 7 and 20 weeks of age (Fig. 1d, h-l, Fig. 3a-d, h, m, Fig. 6f, i-j, ED Fig. 2a, f, g, ED Fig. 4a-h, ED Fig. 6d). Aging cohorts of Galpha13 deficient and littermate control mice were analyzed between 10 and 25 months of age (Fig. 1a-c and ED Fig. 1). Some glutamine supplementation experiments were started on mice that were 8 months old and analyzed after 10 weeks of treatment (Fig. 7g and ED Fig. 7j). All mice were on a C57BL/6 background. Mice were housed in a specific pathogen–free environment (except in gnotobiotic experiments) in ventilated microisolator cages with 12 h light and 12 h dark cycles at 72 F and 40–60% relative humidity. All mouse experiments received approval by the National Cancer Institute Animal Care and Use Committee (NCI-ACUC) and were performed in accordance with NCI-ACUC guidelines and under approved protocol LYMB-001.<br><br>Adult B6-Ly5.1/Cr (B6.SJL-PtprcaPepcb/BoyCrCrl; Stock number 564) mice at least 6 weeks of age were from Charles River Frederick Research Model Facility. Cr2-cre (B6.CgTg(Cr2-cre)3Cgn/J; Stock number 006368), Aicdacre (B6.129P2-Aicdatm1(cre)Mnz/J; Stock number 007770), Rosa26LSL-Cas9 (B6J.129(B6N)-Gt(ROSA)26Sortm1(CAG-cas9*,-EGFP)Fezh/J; Stock number 026175), Rosa26Cas9 (B6(C)-Gt(ROSA)26Sorem1.1(CAG-cas9*,-EGFP)Rsky/J; Stock number 028555) and Mb1-cre (B6.C(Cg)-Cd79atm1(cre)Reth/EhobJ; Stock number 020505) mice were from The Jackson Laboratory. Gna13f/f mice were from S. Coughlin (University of California, San Francisco, San Francisco, CA). S1pr2-creERT2 BAC-transgenic and Rosa26LSL-tdTomato were from T. Okada (RIKEN, Yokohama City, Kanagawa, Japan) |
| Wild animals | This study did not utilize wild animals. |
| Reporting on sex | Male and female mice were used in this study. Littermates that were also sex-matched were used whenever possible to control for co-variates. |
| Field-collected samples | This study did not utilize field-collected samples. |
| Ethics oversight | National Cancer Institute Animal Care and Use Committee (NCI-ACUC). Protocol number: LYMB-001 |

Note that full information on the approval of the study protocol must also be provided in the manuscript.

## Plants

| | |
|---|---|
| Seed stocks | Not applicable |
| Novel plant genotypes | Not applicable |
| Authentication | Not applicable |

# Flow Cytometry

## Plots

Confirm that:

☒ The axis labels state the marker and fluorochrome used (e.g. CD4-FITC).

☒ The axis scales are clearly visible. Include numbers along axes only for bottom left plot of group (a 'group' is an analysis of identical markers).

☒ All plots are contour plots with outliers or pseudocolor plots.

☒ A numerical value for number of cells or percentage (with statistics) is provided.

## Methodology

| | |
|---|---|
| Sample preparation | Mesenteric lymph node, peripheral lymph node or peyer's patch cell suspensions were generated by mashing the organs through 70-mm cell strainers in RPMI containing 2% (v/v) FBS, antibiotics (penicillin (50 IU/ml) and streptomycin (50 mg/ml); Cellgro) and 10 mM HEPES, pH 7.2 (Cellgro). |
| Instrument | Beckman Coulter Cytoflex LX for cell analysis. Sony MA900 for cell sorting. |
| Software | Data was collected using CytExpert v2.3 (Beckman Coulter). Data was analyzed with Flowjo v10 (Treestar) |
| Cell population abundance | In all flow cytometry experiments, 300,000 to 1,000,000 events were recorded per sample.<br>For repertoire sequencing, 8,000 to 42,000 GC B cells were sorted into RLT buffer (Qiagen). GC B cells were sorted as single live cells that were B220+, CD4-, IgDlo, CD38lo, Fas+ and GL7+. For single cell RNA sequencing experiments, ~12,000 cells per sample were sorted into PBS + 0.08% BSA. |
| Gating strategy | Relevant examples of gating strategies are shown in the main and supplemental figures. |

☒ Tick this box to confirm that a figure exemplifying the gating strategy is provided in the Supplementary Information.
