# Supplementary material for: Gα13 restricts nutrient driven proliferation in mucosal germinal centers
Source: Nat Immunol. 2024 Jul 18;25(9):1718–30. doi: 10.1038/s41590-024-01910-0 (PMC11362015; doi:10.1038/s41590-024-01910-0)

Figure 4f

NUDUL1
Myc

75 kDa

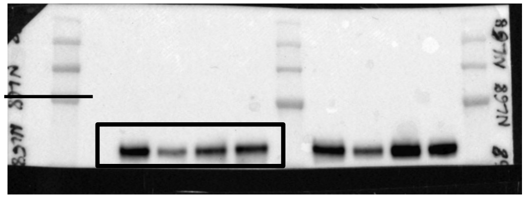

NUDUL1
β-actin

37 kDa

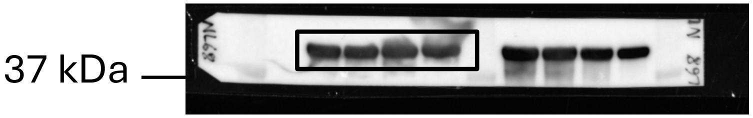

Figure 4f

OCI-Ly8
Myc

75 kDa

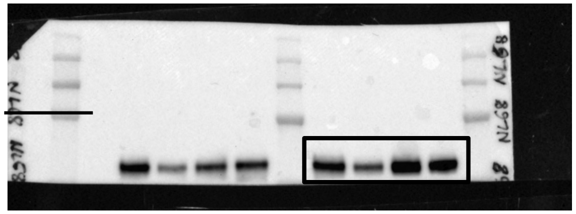

OCI-LY8
β-actin

37 kDa

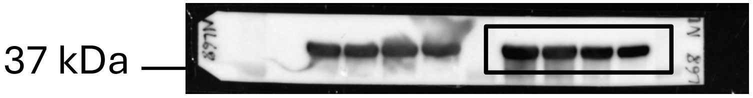

Figure 4f

DOGKIT
Myc

50 kDa

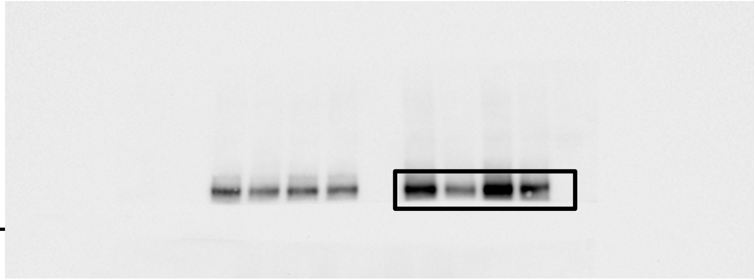

DOGKIT
β-actin

37 kDa

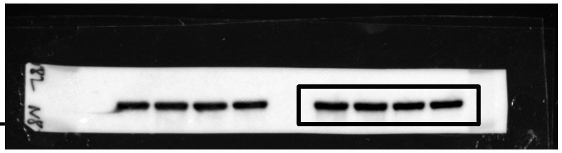

Figure 4f

NUDUL1
CCND3

25 kDa

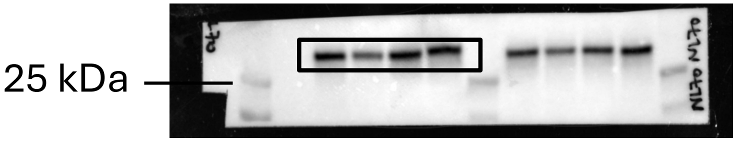

NUDUL1
β-actin

37 kDa

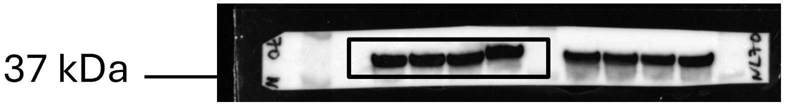

# Figure 4f

OCI-LY8
CCND3

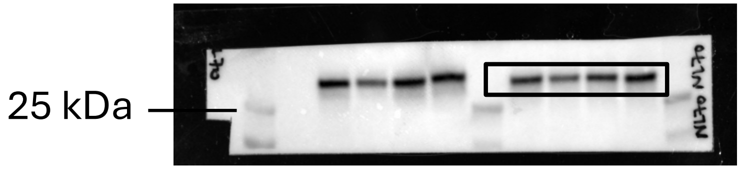


OCI-Ly8
β-actin

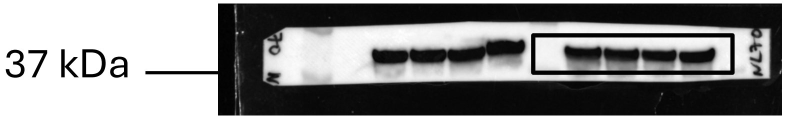

# Figure 4f

DOGKIT
CCND3

25 kDa

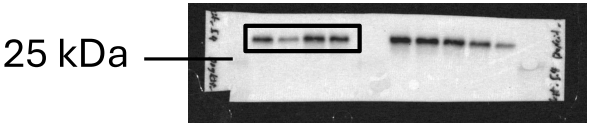

DOGKIT
β-actin

37 kDa

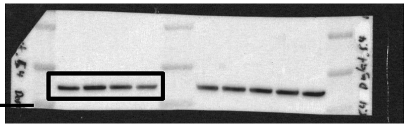

Figure 4g

p-P70S6K T389

75 kDa

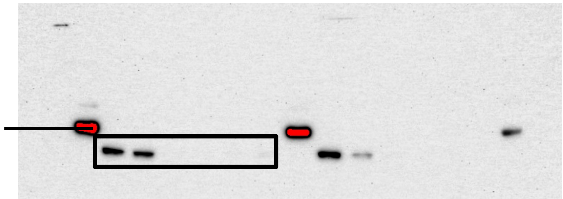

Total P70S6K

75 kDa

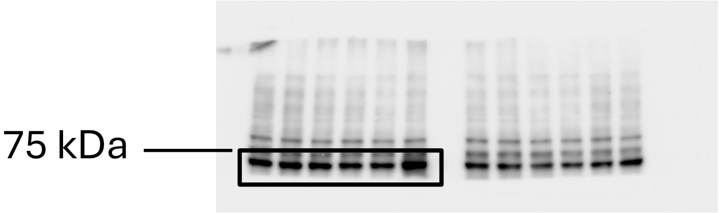

Figure 4g

p-rbS6 S235

25 kDa

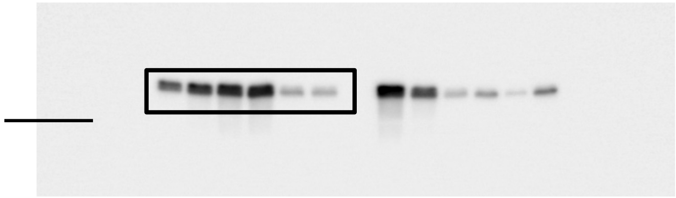

Total rbS6

25 kDa

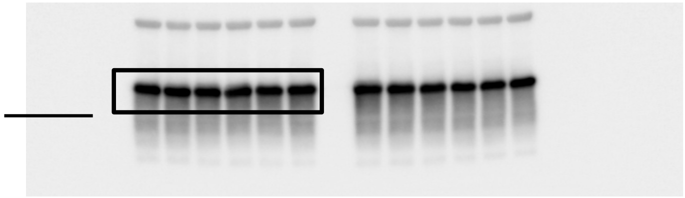

Figure 4g

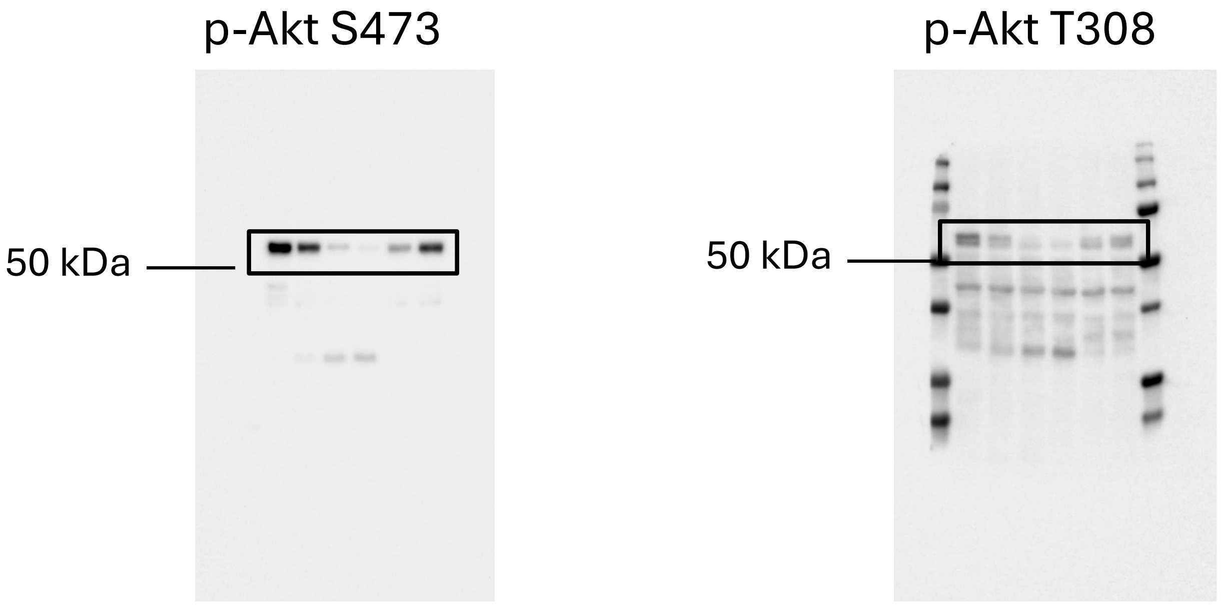

Figure 4g

Total Akt

50 kDa

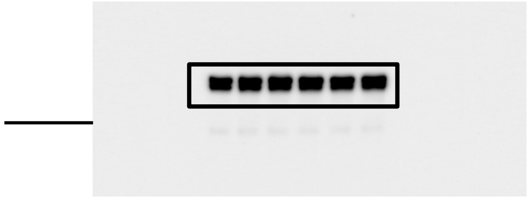

Figure 4g

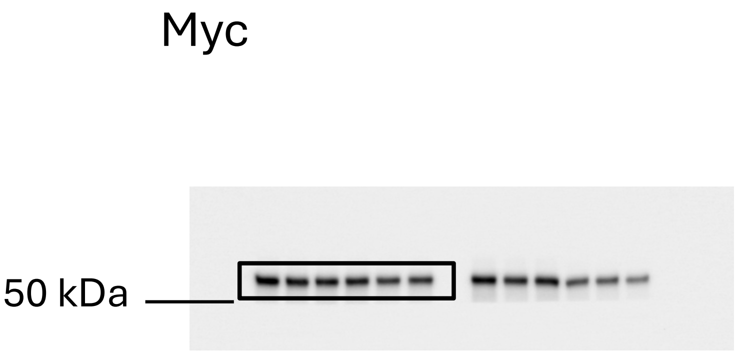


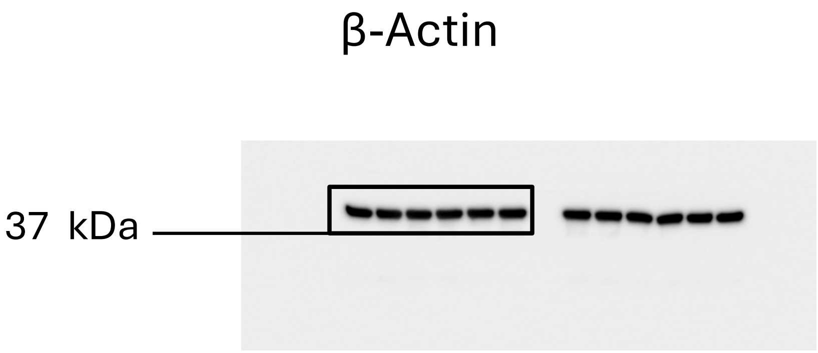

Supplement: Supplementary file 7 — Unprocessed immunoblots. [file 41590_2024_1910_MOESM7_ESM.pdf]
