# Supplementary material for: Gα13 restricts nutrient driven proliferation in mucosal germinal centers
Source: Nat Immunol. 2024 Jul 18;25(9):1718–30. doi: 10.1038/s41590-024-01910-0 (PMC11362015; doi:10.1038/s41590-024-01910-0)

Figure 5b

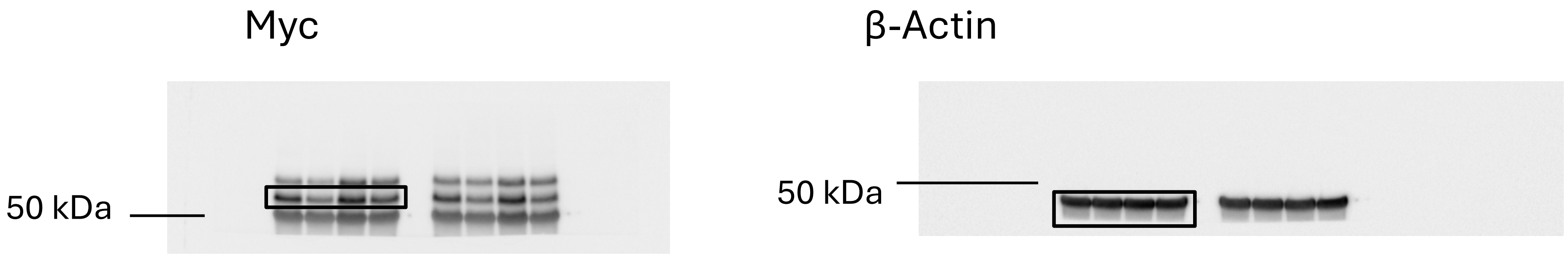

Figure 5c

Myc

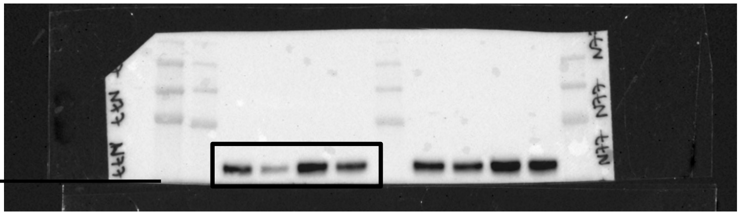

50 kDa

β-Actin

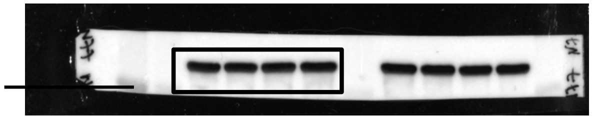

37 kDa

Figure 5d

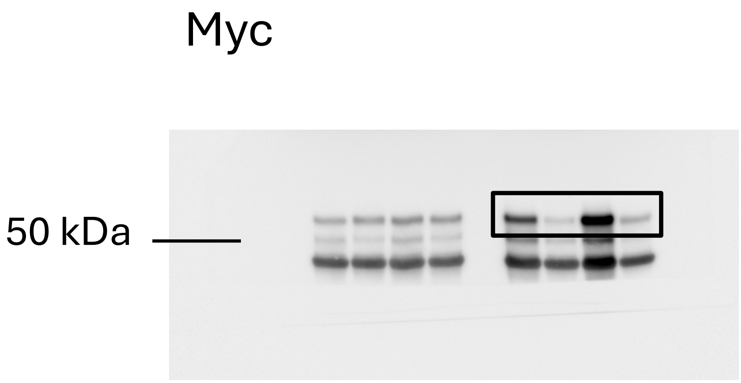

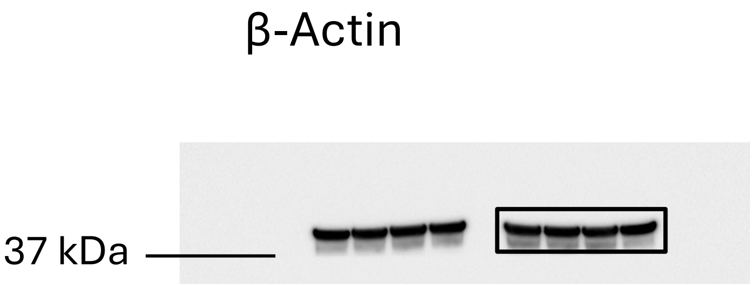

Figure 5e

MYC

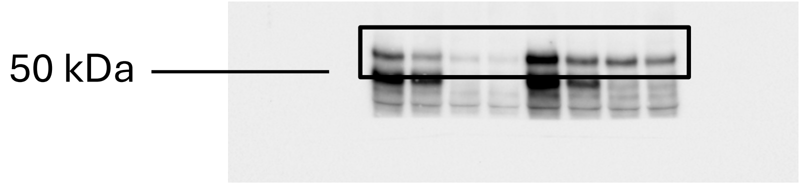

β-Actin

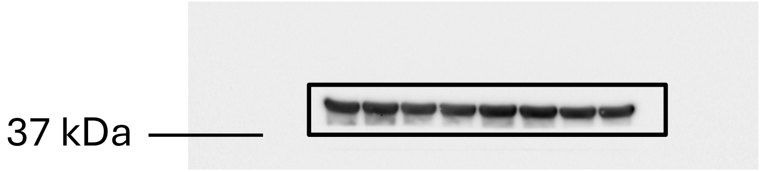

Supplement: Supplementary file 9 — Unprocessed immunoblots. [file 41590_2024_1910_MOESM9_ESM.pdf]
