# Supplementary figures and images for: Gα13 restricts nutrient driven proliferation in mucosal germinal centers
Source: Nat Immunol. 2024 Jul 18;25(9):1718–30. doi: 10.1038/s41590-024-01910-0 (PMC11362015; doi:10.1038/s41590-024-01910-0)

Figure 7a

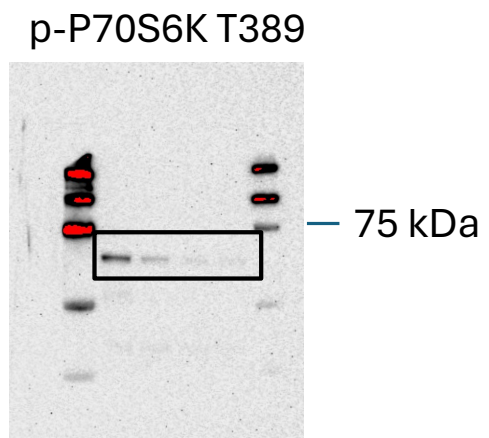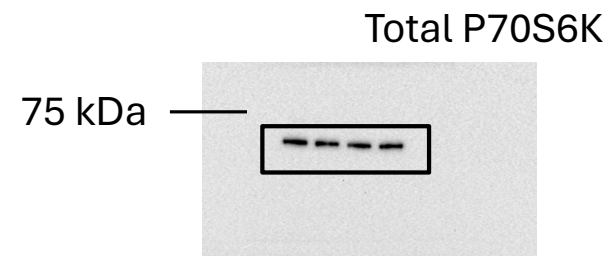

Figure 7a

p-AKT S473

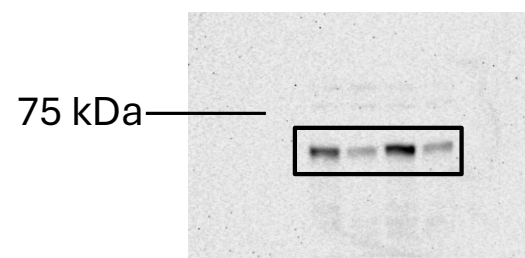

Figure 7a

Myc

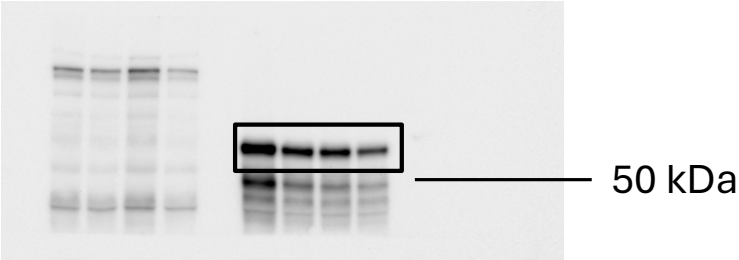

$\beta$ -Actin

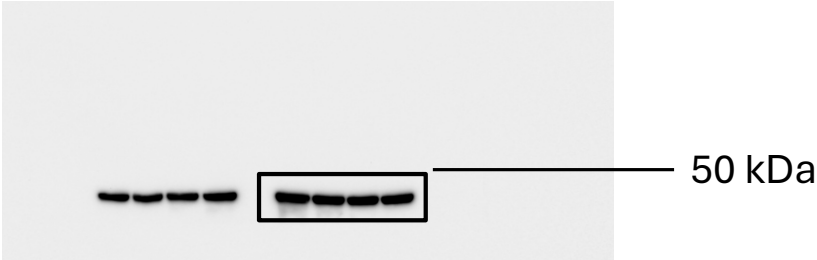

Supplement: Supplementary file 12 — Statistical source data. [file 41590_2024_1910_MOESM12_ESM.pdf]
