# Supplementary material for: Gα13 restricts nutrient driven proliferation in mucosal germinal centers
Source: Nat Immunol. 2024 Jul 18;25(9):1718–30. doi: 10.1038/s41590-024-01910-0 (PMC11362015; doi:10.1038/s41590-024-01910-0)

Extended Data Figure 5e

CCND3

25 kDa

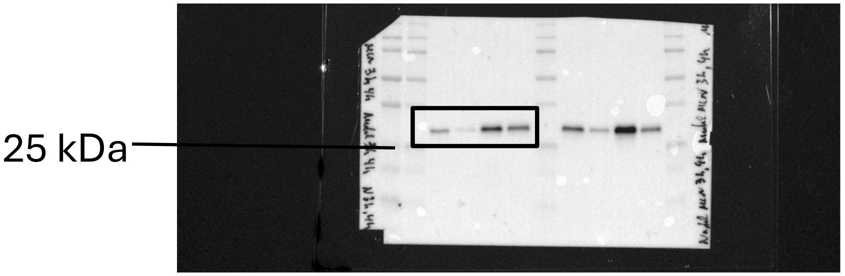

β-Actin

37 kDa

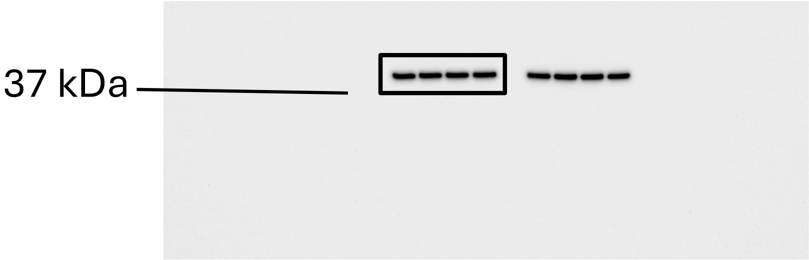

Extended Data Figure 5f

CCND3

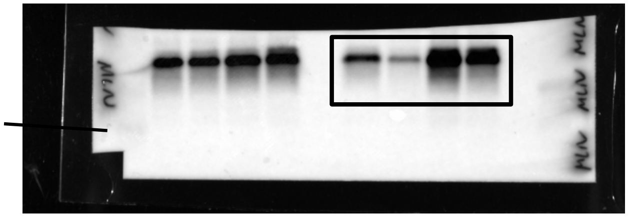

25 kDa

β-Actin

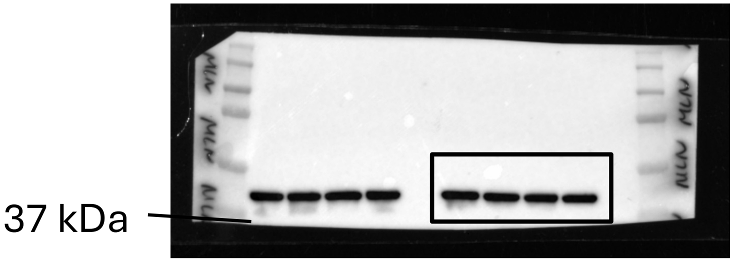

37 kDa

Extended Data Figure 5g

p-Ccnd3 T283

25 kDa

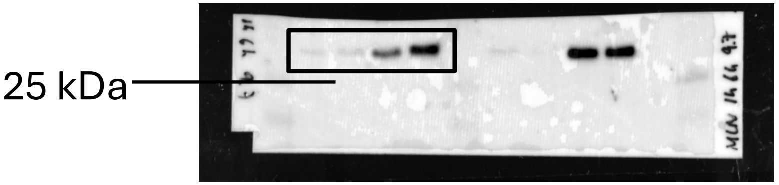

p-Ccnd3 T283

longer exposure

25 kDa

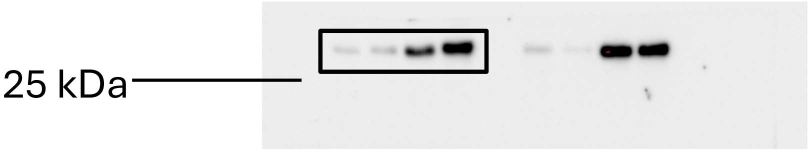

Extended Data Figure 5g

CCND3

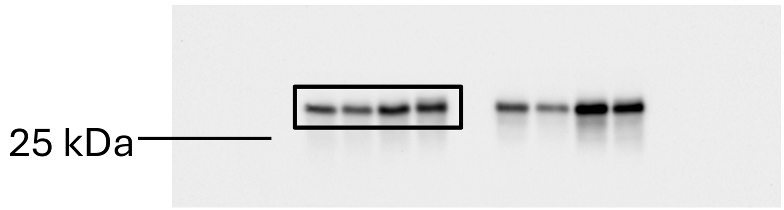

β-Actin

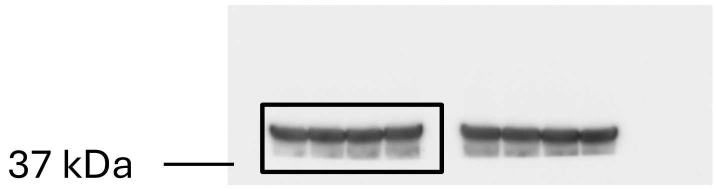

Supplement: Supplementary file 17 — Unprocessed immunolots. [file 41590_2024_1910_MOESM17_ESM.pdf]
